# Volatile Composition, Sensory Profile and Consumer Acceptability of HydroSOStainable Table Olives

**DOI:** 10.3390/foods8100470

**Published:** 2019-10-10

**Authors:** Lucía Sánchez-Rodríguez, Marina Cano-Lamadrid, Ángel A. Carbonell-Barrachina, Esther Sendra, Francisca Hernández

**Affiliations:** 1 Departamento Tecnología Agroalimentaria, Grupo Calidad y Seguridad Alimentaria, Escuela Politécnica Superior de Orihuela, Universidad Miguel Hernández de Elche, Carretera de Beniel, Km 3.2, 03312 Orihuela, Spain; lucia.sanchez@goumh.umh.es (L.S.-R.); marina.cano.umh@gmail.com (M.C.-L.); 2 Departamento Tecnología Agroalimentaria, Grupo Industrialización de Productos de Origen Animal, Escuela Politécnica Superior de Orihuela, Universidad Miguel Hernández de Elche, Carretera de Beniel, Km 3.2, 03312 Orihuela, Spain; Esther.sendra@umh.es; 3 Departamento de Producción Vegetal y Microbiología, Grupo Producción Vegetal, Escuela Politécnica Superior de Orihuela, Universidad Miguel Hernández de Elche, Carretera de Beniel, km 3.2, 03312 Orihuela, Alicante, Spain; francisca.hernandez@umh.es

**Keywords:** bitterness, consumer willingness to pay, descriptive sensory analysis, green-olive flavor, “Manzanilla” cultivar, pit hardening, regulated deficit irrigation

## Abstract

HydroSOStainable table olives (cultivar Manzanilla) are produced from olive trees grown under regulated deficit irrigation (RDI) strategies. Olives produced by RDI are known to have a higher content of some bioactive compounds (e.g. polyphenols), but no information about consumer acceptance (or liking) have been reported so far. In this study, the volatile composition, the sensory profile and the consumer opinion and willingness to pay (at three locations) for HydroSOStainable table olives produced from three RDI treatments and a control were studied. Volatile composition was affected by RDI, by increasing alcohols, ketones and phenolic compounds in some treatments, while others led to a decrease in esters and the content of organic acids. Descriptive sensory analysis (10 panelists) showed an increase of green-olive flavor with a decrease of bitterness in the HydroSOStainable samples. Consumers (study done with 100 consumers in 2-rural and 1-urban locations; *n*_total_ = 300), after being informed about the HydroSOStainable concept, preferred HydroSOStainable table olives to the conventional samples and were willing to pay a higher price for them (52% 1.35–1.75 € and 32% 1.75–2.50 € as compared to the regular price of 1.25 € for a 200 g bag). Finally, green-olive flavor, hardness, crunchiness, bitterness, sweetness and saltiness were defined as the attributes driving consumer acceptance of HydroSOStainable table olives.

## 1. Introduction

Many irrigation treatments have been evaluated in different crops, including olive trees, due to an increasing interest in water-sustainable and environment-friendly products by modern consumers [1,2]. “HydroSOStainable products” are defined for the first time by Noguera-Artiaga et al. [3] as fruits and vegetables cultivated under regulated deficit irrigation (RDI) treatments [3]. Furthermore, Corell et al. [4] have defined HydroSOStainable index for olive trees agronomic conditions. The main aim for application of these types of sustainable strategies is conservation of water (a hot topic in arid farming research) and improving the content of bioactive compounds in vegetables and fruits as a defense mechanism against water stress [5,6,7]. However, to date, the effects of RDI on the consumer acceptability of olives has not been evaluated.

During the last decade, several studies about the effect of RDI on table olives agronomical, chemical and functional characteristics have been published [5,8,9,10,11,12,13], but none of them included consumer insights. The use of moderate RDI (reducing water irrigation in a moderate way but without neglecting irrigation) in table olive orchards led to an enhanced antioxidant capacity and higher polyphenolic content [2,14,15]. Although in those studies, an improvement in the sensory attributes of trees growing under moderate RDI was reported by a trained sensory panel, no consumer acceptance study was conducted. Consumer studies are essential to adjust the sensory profile of food products to consumer demands and needs by adjusting irrigation treatments, to identify the main buying drivers, to develop successful marketing strategies, and to determine an acceptable price for HydroSOStainable table olives. Recently, an affective study carried out in HydroSOStainable almonds [16]; the main conclusion was that RDI strategies led to similar global acceptance than conventional treatments but being sustainable with the environment by saving irrigation water. In addition, consumers were willing to pay a higher price for HydroSOStainable almonds (~2 € kg^−1^ more), which could be an argument to convince farmers to implement these water-saving irrigation technologies. The same behavior was observed in a study with HydroSOStainable pistachios [3], in which authors concluded that consumers were willing to pay approximately 1 euro more per kg of HydroSOStainable pistachio as compared to control samples.

Consequently, the aim of the present study was to evaluate consumer insights about HydroSOStainable table olives produced using different technologies and to link consumer data with descriptive sensory analysis and the contents of the volatile compounds. For that purpose, table olives coming from three RDI treatments [moderate deficit irrigation (T1), severe deficit irrigation during short time (T2) and severe deficit irrigation during long time (T3), and a control were assayed at the field, and the following analyses were conducted: (i) volatile composition by gas-chromatography, (ii) descriptive sensory analysis by a trained panel, and (iii) affective opinion of consumers and their willingness to pay.

## 2. Materials and Methods 

### 2.1. Plant Material and Experimental Design

Olives were collected on September 2017 from a farm, Doña Ana, which is located in Dos Hermanas (Seville, Spain) (37° 25’N, 5° 95’W). Olive trees (cultivar “Manzanilla”) were approximately 32-year-old. Irrigation was performed during the night by drip, using lateral pipes per row of trees and four emitters per plant, split between the two rows (each delivering 2 L h^−1^). A pressure chamber (PMS Instrument Company, Albany, OR, USA) was used to measured stem water potential at midday (*Ψ*_stem_). Water stress integral (SI), calculated as Myers [17] was used to describe the cumulative effect of the water deficit [18]. Three different irrigation treatments and a control were carried out:

- control (T0), trees were fully irrigated, to avoid any water stress;
- moderate deficit irrigation (T1), the threshold value for water stress level (*Ψ*_stem_) was set up at −2 MPa during pit hardening stage;
- severe deficit irrigation (short time) (T2), the threshold value for *Ψ*_stem_ was set up at −3 MPa during half period of pit hardening stage; and,
- severe deficit irrigation (long time) (T3), the threshold value for *Ψ*_stem_ was −3 MPa until the end of the period of pit hardening stage.

Table 1 shows the average of minimum stem water potential (min *Ψ*_stem_) and SI values, together with the volume of applied water in each treatment.

### 2.2. Spanish-style Processing

For each RDI treatment, four batches of fresh olives were processed. Each one was formed by 50 kg of raw olives that were mixed and transported to Cooperativa Nuestra Señora de las Virtudes (La Puebla de Cazalla, Seville, Spain). First, olives were submitted to lye treatment during 6–8 h with 1.3–2.6% (weight:volume) of NaOH. Then, olives were washed with water during 12 h for cleaning and they were put on 12% NaCl for fermentation (it began with 0.17 mol L^−1^ and finished with 0.09 mol L^−1^). After 4 months of fermentation, table olives reached an equilibrium with brine (pH < 4.2, 8% NaCl, 0.8% lactic acid and residual alkalinity < 0.120 N).

### 2.3. Volatile Compounds

Volatile extraction was performed using headspace solid phase micro-extraction (HS-SPME). Analysis were carried out according to Cano-Lamadrid et al. [2]. Briefly, 5 g of olives mixed with 15 mL of ultrapure water and 1.5 g of NaCl were placed into a vial. The vial was put in a bath at 40 °C and, after equilibration, a 50/30 µm divinylbenzene/carboxen/polydimethylsiloxane fiber (2 cm, 24 ga, StableFlex) was manually exposed to the headspace during 50 min. Volatiles were desorbed from fiber into the Gas Chromatograph-Mass Spectrometry (GC-MS) for 3 min.

V+olatile compounds identification was performed in a gas chromatograph, Shimadzu GC-17A (Shimadzu Corporation, Kyoto, Japan), coupled with a Shimadzu mass spectrometer detector GC-MS QP-5050A. GC-MS was equipped with a Restek Rxi-1301 2016 column. Helium was used as carrier gas with same program previously reported by Cano-Lamadrid et al. [2]. Identification was based on: (i) retention indices, (ii) GC-MS retention times, and (iii) mass spectra matches in Wiley 09 MS library (Wiley, New York, NY, USA) and NIST14 (National Institute of Standards and Technology, Gaithersburg, MD, USA). Results for each of the volatile compounds were expressed as percentage of the total area. 

### 2.4. Sensory Analysis

#### 2.4.1. Descriptive Sensory Evaluation

Ten trained panelists (aged from 25–55 years) from the Food Quality and Safety research group (Miguel Hernández University of Elche, Alicante, Spain) carried out the descriptive sensory analysis of samples under study. Each panelist had more than 600 h of experience with a variety of products, mostly, vegetable or horticultural products. For the present study, the panel was trained during 3 sessions of 1 h each, where they worked on the International Olive Oil Council, IOOC [19] table olives lexicon and finally, the panel agreed on the useful lexicon for the samples: color (from yellow to green), saltiness, bitterness, sourness, sweetness, aftertaste, hardness, crunchiness and fibrousness, and off-flavors or negative attributes; if off-flavors were present panelists could choose among the options abnormal fermentation, musty, rancid, cooking effect, soapy, metallic, earthy, and winey-vinegary [19].

Odor-free disposable 100 mL plastic cups were used to serve samples to panelists at room temperature (~20 °C). Cups were half filled with table olives coded with random 3-digit numbers and covered. Distillated water and crackers were used to cleanse palates between samples. Three sessions were used for the descriptive sensory evaluation of samples (each sample was evaluated in triplicate). Panelists used a 0–10 scale (0: no intensity; and 10: extremely strong).

#### 2.4.2. Consumer Acceptance

For affective sensory evaluation, 100 regular table olive consumers were invited from three locations: (i) L1: El Esparragal (Murcia, Spain); (ii) L2: Elche (Alicante, Spain); and, (iii) L3: Los Desamparados (Alicante, Spain). L1 and L3 were chosen to represent consumers from rural areas, while L2 was chosen to represent consumers from urban locations. Consumers were recruited by telephone from the database of SensoFood Solutions of Universidad Miguel Hernández de Elche. The eligibility criteria was that they consume, at least, three times per week table olives. Informed consent was obtained and it is available from the Principal Investigators of the project AGL2016-75794-C4-1-R, Prof. Carbonell-Barrachina. Demographic questions were added to the questionnaire. The consumer age range was 18–24 (13%), 25–35 (14%), 36–45 (19%), 45–55 (26%) and >55 (28%) with a 62:38 gender ratio (women:men). Forty-six percent of consumers participating in this study were full-time workers, 17% part-time, 17% were students and 20% were unemployed. Consumers were also asked about their interest on food labels, and 79% answered that pay attention to product labels, especially, for Spanish-products (64%), healthy products (57%) and sustainable products (25%).

The study was carried out using SensoFood Solutions individual booths (Inverso Estudio Creativo, Murcia, Spain) in all locations to isolate participants and ensure that they worked individually, with a randomized block design and using 3-digits codes for each sample. Samples were served following the same way as for descriptive sensory evaluation. Questionnaires were prepared using 9-point hedonic scale (1 = dislike extremely, 5 = neither like nor dislike, and 9 = like extremely) for color, flavor, bitterness, saltiness, sourness, hardness, crunchiness, fibrousness, aftertaste and overall. Just About Right (JAR) scale (1 = low intensity, and 9 = high intensity) was also used to score intensity attributes (flavor, bitterness, saltiness, sourness and aftertaste) to later evaluate how samples could be improve using penalty analysis. Additionally, preference test was done to rank irrigation treatments under study where consumers had to order table olive samples from dislike to like and later, Friedman test was carried out to interpret data.

All panelists (descriptive test) and consumers (affective tests) gave their informed consent for inclusion before they participated in the study. Universidad Miguel Hernández de Elche automatically exempts “general taste tests”, including descriptive sensory tests from needing ethical approval, based on European Union guidelines. However, the study was conducted in accordance with the Declaration of Helsinki, and the protocol was approved by the Ethics Committee of the Escuela Politécnica Superior de Orihuela, Universidad Miguel Hernández de Elche (project AGL2016-75794-C4-1-R).

#### 2.4.3. Consumer Willingness to Pay

**Figure 1:**
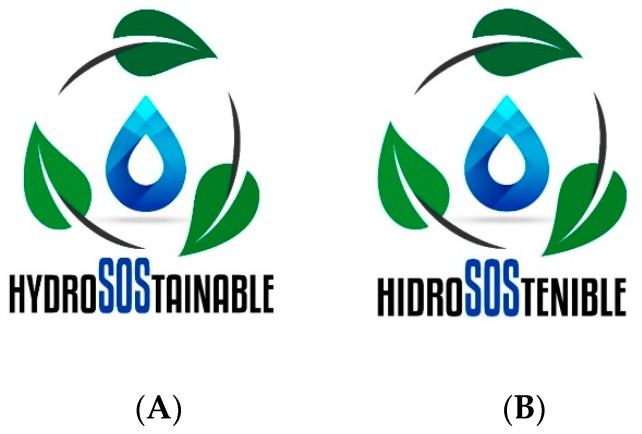
HydroSOStainable logo. (**A**): English version. (**B**): Spanish version.

Consumer were first informed about HydroSOStainability concept by a leaflet and answering their questions. Then, two samples of table olives were provided to them. Commercial Spanish-style “Manzanilla” table olives were purchased from Mercadona supermarket (Mercadona is one of the most popular food supermarkets in the Mediterranean area of Spain). These table olives were labeled as “conventional” as opposed to olives labeled “HydroSOStainable”, with its logo (Figure 1); in this way, the same product was presented to the consumers but with and without the HydroSOStainability logo. Each sample (“conventional” or “HydroSOStainable”) was presented to the consumer together with its corresponding questionnaire. Firstly, consumer evaluated “conventional” table olives green-olive flavor, saltiness, hardness and overall liking, and secondly, HydroSOStainable table olives green-olive flavor, saltiness, hardness overall liking and willingness to pay. They were given a price for conventional table olives of 1.35 € per 200 g (Mercadona price) and 4 options to pay for HydroSOStainable table olives: ≤1.35 € (distributor brand), range 1.35–1.75 € (known brand prices), range 1.75–2.50 € (known brand prices), and >2.50 € (gourmet table olives).

This study was done in the same three locations than the affective sensory evaluation but using 100 consumers in each site (some of them were the same than in the affective sensory evaluation). 

### 2.5. Statistical Analysis

Two or three-way analysis of variance (ANOVA) followed by Tukey’s multiple range test were the chosen statistical tests. To assess panel performance, a 3-way ANOVA (factor 1: irrigation treatment; factor 2: panel session; and, factor 3: panelist) was carried out in the descriptive sensory evaluation. For affective sensory data, 2-way ANOVA was used (factor 1: irrigation treatment; and, factor 2: location). Additionally, penalty analysis was carried out with JAR data from the affective test to study how samples could be improved, and partial least squares regression (PLS) was also performed to correlate consumer overall liking with the volatile compounds and descriptive sensory attributes. All statistics were performed using XLSTAT Premium 2016 (Addinsoft, New York, NY, USA). Finally, data from the JAR analysis (Penalty analysis) were graphically represented.

## 3. Results and Discussion

### 3.1. Irrigation

Table 1 summarizes the information regarding the water stress achieved by the olive trees during 2017 season, by using 2 parameters (minimum midday stem water potential (min *Ψ*_stem_) and water stress integral (SI)). Statistical differences were found among three RDI treatments and control in both parameters studied, Min *Ψ*_stem_ and SI. In fact, T3 was the treatment presenting the highest SI value (69.2 MPa × day) as well as the highest min *Ψ*_stem_ (−3.69 MPa) and this strong stress was basically due to the fact that the smallest volume of water was applied (105.1 mm). T1 and T2 occupied an intermediate position, reflecting a moderate water stress level as compared to T0 (control), which trees suffered the lowest stress. T1 and T2 were not statistically different although the stress applied was different (harder for T2) because of time of application, so applying moderate stress during log time and severe stress during short time caused similar stress on trees. These results followed a similar trend to those from previous seasons (2015 and 2016), as reported by Sánchez-Rodríguez et al. [18].

### 3.2. Volatile Compounds

Thirty-eight volatile compounds were identified in the table olives and their content for each irrigation treatment are shown in Table 2. Esters were the predominant volatiles in control table olives (38.48%), although their content decreased as RDI was more severe. On the contrary, terpenes were the predominant chemical family on HydroSOStainable table olives (T1–T3), with T2 olives (severe deficit irrigation, short time) having the highest content (47.39%). Organic acids were also in a high proportion (>10%) in all table olives, except T2 (2.95%). Besides, T2 showed the highest percentage of ketones (14.47%), while phenolic compounds and alcohols having similar contents in T1 and T3 samples but higher than those of T0 and T2.

There are some volatile compounds that showed the same trend in all RDI table olives, such as ethyl acetate, isoamyl acetate, *cis*-3-hexen-1-ol, 1-hexanol and γ-terpineol, that increased when water stress was applied, and, therefore, HydroSOStainable table olives would have, at least theoretically, stronger pineapple, banana, pear, green, woody and lilac notes than control samples. On the other hand, other compounds showed a decreased content when RDI treatments were applied (2-butanol, propanoic acid, ethyl cyclohexanecarboxylate and cyclohexanecarboxylic acid, butyl ester). Apart from these general trends, T1 experienced an increase on the contents of ethanol, dimethylsulfide (green, sulfurous), acetic acid (vinegar), ethyl propionate (fruity, pineapple), n-propyl acetate (celery), propyl propionate (oily, fruity), propyl butanoate and p-cresol (green, woody). With respect to T2, dimethylsulfide, propyl butanoate, D-limonene (citrus, lemon), p-cymene (citrus), γ-Terpinene (herbaceous, citrus), ethyl propanoate (fruity, melon, peach) and 6-methyl-5-hepten-2-one (herbaceous, oily) as compared to the control table olives, while 2-butanol, acetic acid and p-cresol were not found on these samples. Finally, T3 olives had an increased content of ethyl heptanoate, guaiacol (woody, smoky) and cyclohexanecarboxylic acid (fatty, fruity) but a decreased content on 2-butanol, propyl propionate and p-cresol always as compared to control samples. The sensory descriptors were obtained from relevant olive related references, including GC-olfactometry studies [2,20].

A previous study with “Manzanilla” Spanish-style table olives processed in the same way than in the current research, but under different irrigation conditions also showed statistically significant differences in a high number of volatile compounds [2]. For instance, it was found that acids and straight chain hydrocarbons increased their concentration simultaneously with the stress while aldehydes and phenol compounds decreased. These results did not agree with those found in the current research but it could be due to different irrigation conditions, among other agronomic differences such as soil characteristic or climate conditions. Brahmi, et al. [21] also found differences among volatile compounds as affected by the irrigation strategies on “Koroneiki” cultivar grown under Tunisian conditions. The content of some alcohols decreased, but others increased as it was found in the present work. In the same way, it was found that some aldehydes decreased.

### 3.3. Descriptive Sensory Analysis

Descriptive sensory analysis by trained panel (0–10 scale) of table olives under study was carried out and results are shown in Table 3. Saltiness, sweetness and fibrousness had mean values (for all treatments under study) of 5.4, 2.2 and 0.5, respectively; no statistically significant (ANOVA, *p* < 0.05) differences were found for these attributes and mean values are reported. With respect to color, T0 olives presented the highest color intensity (6.5), while T1 had the lowest intensity (5.4), and therefore the most yellowish color. T2 and T3 showed intermediate positions and thus, they presented intermediate colors between yellow and green. As far as the green-olive flavor is concerned, T1 table olives had the highest intensity (6.9), with T3 having the lowest score (6.2), and T0 and T2 having being in the middle. Bitterness decreased its intensity (up to 3 points) as the water stress increased. The T3 olives were the sourest ones (4.5 points higher than control) and at the same time had the longest aftertaste (2.2 points higher than control), but they simultaneously had the lowest intensity of hardness and crunchiness (3.5 and 1.7, respectively). Finally, it is important to mention that no off-flavors were found in any of the table olive under study.

Previous studies had also found changes on the intensity of key sensory descriptors as an effect of irrigation regimes on table olives. For instance, Cano-Lamadrid et al. [2] and Cano-Lamadrid et al. [13] showed the effect of two RDI treatments on the descriptive sensory profile of “Manzanilla” Spanish-style table olives. In those studies, saltiness, green-olive flavor, aftertaste, bitterness and hardness were affected by irrigation. It was found that moderate stress caused an increase of ~5% on the intensity value of the green-olive flavor attribute; result which agreed well with the trend just reported on the current research. However, results on bitterness and aftertaste showed an increase in trees grown under moderate stress [2] while in the current experiment a decreased intensity of bitterness and aftertaste (as compared to the control sample) at moderate level, while an increased aftertaste intensity was observed at severe stress. With respect to bitterness, a similar result was found on “Ascolana” olives [5], in which the bitter character decreased with the irrigation regime. The same trend was also found for hardness [5], which agreed with the low hardness of the T3 samples in the present work. In the case of “Nocellara del Belice” cultivar produced following Greek style [13], an increase on green-olive aroma, sourness, sweetness and crispness were reported under moderate water stress.

### 3.4. Consumer Acceptance

Affective sensory evaluation was carried out at three locations, although no statistical differences were found among data obtained; thus, the mean values of nine descriptors and the corresponding overall liking of consumers at the three locations is shown in Table 4. Table olives showed a high overall acceptability by consumers (mean of 6.3 in a scale up to a maximum score of 9). The rest of attributes under study (color, 6.5; flavor, 6.4; bitterness, 6.0, saltiness, 6.1; sourness, 6.0; hardness, 6.6; crunchiness, 6.6; fibrousness, 6.5; and aftertaste, 6.2) also received high values (1–9 scale) of consumer satisfaction degree.

Consumer preference for table olives was analyzed using the Friedman test. No statistical significant differences (*p* < 0.05) were found among preferences for control (T0) and HydroSOStainable table olives (T1–T3). Thus, this experimental finding confirmed that HydroSOStainable olives were as least as preferred as those coming from fully irrigated trees (T0), but saving water and being more sustainable; this sustainability makes these olives attractive for consumption [23].

From the best of our knowledge, only one affective sensory evaluation had been previously conducted for table olives coming for RDI treatments [2]. In this study, “Manzanilla” Spanish-style table olives under moderate deficit irrigation (but with different treatments than in the current research) were the preferred ones by consumers because of their flavor, crunchiness and aftertaste.

### 3.5. Driving Sensory Attributes

**Figure 2:**
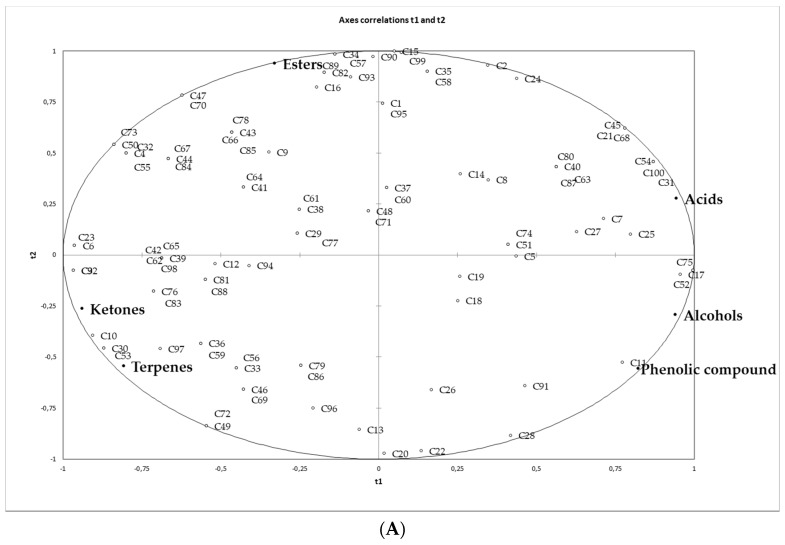

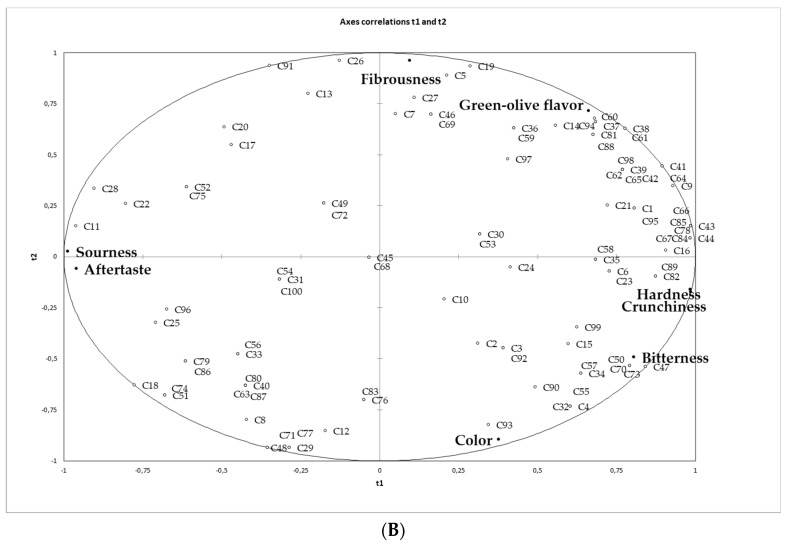
Partial least squares regression (PLS) of (**A**) volatile compounds (chemical families sum) (X axis: t2) and overall consumer liking (Y axis: t1) (unfiled circles: consumer (C + number of consumer); filled circle: volatile compound); and, (**B**) descriptive sensory attributes (X axis) and overall consumer liking (Y axis) (unfiled circles: consumer (C + number of consumer; filled circle: descriptor).

PLS Regression analysis was carried out to established drivers of liking for HydroSOStainable table olives (Figure 2). Two PLS maps were constructed to correlate the consumer overall liking (affective sensory analysis) with volatile compounds (total volatile contents for each chemical family) (Figure 2A) and with descriptive sensory attributes (trained panelists) (Figure 2B). Only attributes showing statistical differences among samples (ANOVA *p* < 0.05) were used to construct maps.

In the positive part of the x-axis (right side of the graph) volatiles associated with overall liking of consumers were acids, alcohols and phenolic compounds while in the negative part of the x-axis, ketones and terpenes can be found (Figure 2A). Although these volatile families are in opposite places on the map, consumer overall liking were not concentrate in any specific part of the map as a high dispersion on the map could be found; thus, it was not stated that no a clear relationship between overall consumer liking (affective sensory analysis) and volatile compounds was observed. Therefore, volatiles could not be considered as good driving sensory attributes for the acceptability of HydroSOStainable table olives. 

Regarding map B (Figure 2B), consumer satisfaction (affective sensory analysis) was correlated with some positive attributes (descriptive sensory analysis by trained panel) of table olives such as green-olive flavor, hardness, crunchiness and bitterness, as it can be observed a high concentration of consumer overall liking in the right side of the map, where these descriptors are positioned. Consequently, these descriptors should be use as drivers to understand future consumer acceptance of HydroSOStainable table olives. 

### 3.6. Consumer Willingness to Pay

Table 5 shows the results of overall liking and satisfaction degree study done regarding consumer willingness to pay for table olives at three locations. Green-olive flavor, saltiness, hardness and consumer overall liking were evaluated as the most important attributes valued by consumers to further understanding on their perception of HydroSOStainable logo. This logo (Figure 1), caused a clear effect on consumer overall liking and green-olive flavor perception, making HydroSOStainable samples to increase their values in 1.1 and 1.3 units, respectively, as compared to the control olives. Concerning the location, for green-olive flavor attribute, consumers in L1 punctuated olives with the highest score (7.7) while L2 with the lowest (7.0), but the opposite occurred for overall liking, where L2 scored with the highest satisfaction degree (7.3). Regarding the interaction logo and location, the highest scores of the green-olive flavor attribute were found in L1 and L3 samples with the HydroSOStainability logo, and the lowest values was found in the L3 table olives without the HydroSOStainability logo. It is important to consider that L2 consumers (Elche, Alicante, Spain), corresponding to people living in an urban location, scored the highest for the overall liking without any need for the hydroSOStainability logo. No significant statistical differences were found for the effects of logo, location and their interaction on table olives saltiness and hardness.

Regarding willingness to pay, 88% of the participants in the study were willing to pay more than the usual price (1.35 € per 200 g) when they were informed about HydroSOStainable benefits. Concretely, 52% were willing to pay a price in the range 1.35–1.75 €, 32% 1.75-2.50 € and only 4% were willing to pay more than 2.50 €.

Previous study done with HydroSOStainable pistachios [3] also reported an increase of willingness to pay. In that case, the study was conducted in Galicia (northern Spain) and the Valencian Community (representing Mediterranean area of Spain) and consumers from Galicia willing to pay more than those from the Valencian Community; although all consumers agreed that the price for this product should be higher than for the conventional ones. A similar situation was reported by Lipan et al. [16], where Spanish and Romanian consumers were willing to pay more for HydroSOStainable almonds.

### 3.7. Penalty Analysis

**Figure 3:**
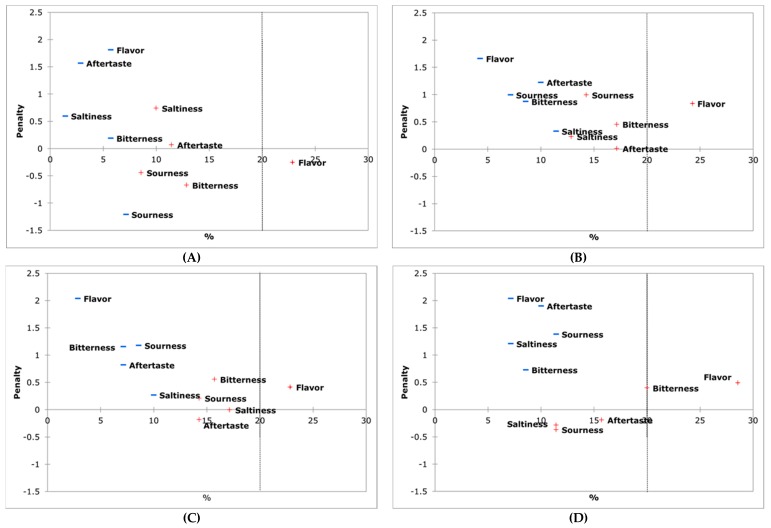
Penalty analysis of samples (**A**) = T0; (**B**) = T1; (**C**) = T2; (**D**) = T3. “Too low intensity” is indicated with “−“ and “too high intensity” is indicated with “+”.

Apart from the above described overall liking and satisfaction degree for specific sensory attributes, several JAR questions (flavor, bitterness, saltiness, sourness and aftertaste) were asked along the consumer study (affective sensory evaluation) with the purpose of analyzing the possible intensity attributes to be improved. Penalty analysis was conducted [24] an easier understanding of the relationship between JAR scores and consumer satisfaction degree scores. Figure 3 shows the proportion of consumer opinion plots against the mean penalty score. The attributes susceptible of improvement were those, which had the greatest negative impact on the sample liking for at least 20% of consumers and caused a drop of at least 1 point for liking. Results of the penalty analysis indicated that the studied deficit irrigation treatments (T1, T2 and T3) were not penalized by presenting low or high intensities of the studied attributes (Figure 2B–D). According to Spanish consumers, no improvement was necessary in these olive samples.

Previous research about overall consumer liking of HydroSOStainable almonds [16] results indicated that only the bitterness could be improved (decreasing it) when “sustained” deficit irrigation treatment was applied (deficit irrigation during whole season); however, when using RDI, HydroSOStainable almonds did not show any attribute to be improved, as it was found here for HydroSOStainable table olives, so this treatments were the best for consumer acceptance as their quality was as high as control table olives.

## 4. Conclusions

This is the first study about consumer acceptance and willingness to pay for table olives under RDI treatments (HydroSOStainable table olives). Results indicated that RDI produced changes on volatile composition and on the intensity of several sensory descriptors. Green-olive flavor, hardness, crunchiness and bitterness seem to be the driving sensory attributes controlling consumer acceptance for HydroSOStainable table olives, although further studies are needed to fully prove this statement. Consumers preferred table olives with the HydroSOStainability logo and their satisfaction level was higher for the green-olive flavor and overall liking as compared to those of the conventional samples (without this logo). A high percentage of consumers were willing to pay a higher price for HydroSOStainable table olives. Information obtained in this research should be useful for developing the best irrigation strategy to produce table olives with the highest water saving, and the best sensory characteristics for consumers. For instance, T1 (moderate deficit irrigation where *Ψ*_stem_ was −2 MPa during pit hardening stage) and T2 (severe deficit irrigation during short time where *Ψ*_stem_ was −3 MPa during half period of pit hardening stage) strategies optimized for desirable sensory characteristics, such as green-olive flavor, hardness and crunchiness.

## Figures and Tables

**Table 1:** Minimum midday stem water potential (min *Ψ*_stem_), water stress integral (SI) and water applied as affected by the irrigation treatment.

Sample	Min *Ψ*_stem_ (MPa)	SI (MPa × Day)	Water Applied (mm)
**ANOVA** ^†^			
	*	**	NS
**Multiple Range Tukey Test** ^‡^			
T0	−2.16 ^a^	17.5 ^b^	274.3
T1	−3.07 ^b,c^	45.4 ^a,b^	294.9
T2	−2.44 ^a,b^	31.3 ^a,b^	347.7
T3	−3.69 ^c^	69.2 ^a^	105.1

† NS = not significant at *p* > 0.05. * and ** significant at *p* < 0.05, and 0.01, respectively. ‡ Values followed by the same letter within the same column were not significantly different (*p* > 0.05), according to Tukey’s least significant difference test.

**Table 2:** Retention indexes, sensory descriptors and percentage of total area of volatile compounds found in table olives as affected by the irrigation treatment.

Compounds	Chemical Family	Ions	RI		Descriptors ^§^	ANOVA ^†^	Content (%)			
		m/z	Exp.	Lit.			T0	T1	T2	T3
Ethanol	Alcohol	45	659			**	0.663 ^b,‡^	1.135 ^a^	0.604 ^b^	0.998 ^a,b^
Dimethylsulfide	Sulfur compound	62/47	679		Green, sulfurous	*	0.221 ^c^	0.552 ^b^	1.063 ^a^	0.285 ^c^
Ethyl acetate	Ester	45/61/70/88	703		Pineapple	**	1.243 ^c^	1.856 ^b^	2.319 ^a^	2.115 ^a,b^
2-Butanol	Alcohol	45	704			*	0.690 ^a^	0.430 ^a,b^	nd ^c^	0.285 ^b^
Acetic acid	Acid	45/60	724		Vinegar	***	11.86 ^b^	14.11 ^a^	nd ^c^	11.03 ^b^
Ethyl propionate	Ester	57	746	726	Fruity, pineapple	*	0.953 ^b,c^	1.764 ^a^	1.377 ^b^	0.737 ^c^
n-Propyl acetate	Ester	61/73	749	728	Celery	*	1.105 ^b,c^	2.040 ^a^	1.353 ^b^	0.927 ^c^
Propanoic acid	Acid	74/45	771		Dairy, acidic	*	0.925 ^a^	0.614 ^b^	0.217 ^c^	0.238 ^c^
2,4-dimethylhexane	Hydrocarbon	85/57/71	793			NS	0.580	1.135	0.773 ^b^	0.523
Ethyl butanoate	Ester	71	812	802		NS	0.221	0.706	0.411	0.333
Propyl propionate	Ester	57/75	820	810	Oily, fruity	*	1.022 ^b^	1.595 ^a^	1.208 ^b^	0.713 ^c^
Butyl acetate	Ester	56/73	827	812	Fruity, greenish	NS	0.041	0.184	0.121	0.166
Ethyl lactate	Ester	45	846	813	Butter, fruity	NS	0.083	0.230	0.121	0.095
Ethyl 2-methyl butanoate	Ester	57/102/85	861	846		NS	0.124	0.368	0.242	0.190
Ethyl 3-methyl butanoate	Ester	88/57	865	859		NS	0.124	0.199	0.145	0.166
Isoamyl acetate	Ester	55/70	895	878	Banana, pear	*	0.041 ^c^	0.138 ^a^	0.072 ^b^	0.048 ^a^
*cis* 3-Hexen-1-ol	Alcohol	67/55/82	899	902	Green	***	0.097 ^c^	0.245 ^a^	0.121 ^b^	0.119 ^b^
1-Hexanol	Alcohol	56/69	907	912	Green, woody	**	0.069 ^c^	0.153 ^a^	0.097 ^b^	0.143 ^a^
Propyl butanoate	Ester	71/89/55	914	896		*	0.152 ^c^	0.629 ^a^	0.362 ^b^	0.119 ^c^
β-Myrcene	Terpene	93/69	997	992	Fruity, vegetable	***	0.801	1.089	1.594	1.426
Ethyl hexanoate	Ester	88	1016	1001		NS	1.229	2.086	2.126	1.949
D-Limonene	Terpene	68/93	1041	1044	Citrus, lemon	***	20.97 ^b^	20.92 ^b^	34.44 ^a^	21.17 ^b^
*p*-Cymene	Terpene	119/134/91	1044	1030	Citrus	**	3.148 ^c^	3.896 ^b,c^	6.449 ^a^	4.705 ^b^
*γ*-Terpinene	Terpene	93/91/136	1069	1076	Herbaceous, citrus	**	2.223 ^b^	2.470 ^b^	3.913 ^a^	2.733 ^a,b^
Methyl cyclohexanecarboxylate	Ester	55/87	1093	1056	Berry, creamy	NS	5.633	2.807	1.957	3.446
Ethyl heptanoate	Ester	88/115/60	1117	1095	Fruity, melon, peach	***	0.690 ^b^	0.890 ^b^	2.101 ^a^	2.163 ^a^
Guaiacol	Phenolic compound	109/124/81	1148	1114	Woody, smoky	***	0.318 ^b^	0.322 ^b^	0.725 ^b^	18.560 ^a^
Ethyl cyclohexanecarboxylate	Ester	55/83/101	1163	1170		***	25.81 ^a^	8.943 ^c^	10.72 ^b^	2.614 ^d^
*p*-Cresol	Phenolic compound	107	1180		Green, woody	***	2.844 ^b^	12.62 ^a^	nd ^c^	0.285 ^c^
2-Phenethylalcohol	Alcohol	91/107	1184	1159	Honey, rose	*	0.207	0.675	0.411	1.355
Cyclohexanecarboxylic acid	Acid	56/73/45/82	1197	1157	Fatty, fruity	**	0.801 ^b^	0.123 ^b^	nd ^b^	10.91 ^a^
6-Methyl-5-hepten-2-one	Ketone	55/108/69/91	1207		Herbaceous, oily	**	3.907 ^b,c^	6.412 ^b^	14.469 ^a^	0.974 ^c^
*γ*-Terpineol	Terpene	59/93/121/136	1243	1224	Lilac	*	0.400 ^c^	0.660 ^b^	0.990 ^a,b^	1.972 ^a^
1,4-Dimethoxy-benzene	Phenolic compound	123/138/95	1254		Fatty	**	2.968 ^c^	5.093 ^a^	5.217 ^a^	4.111 ^b^
Cyclohexanecarboxylic acid, butil ester	Acid	129/83/55/111	1266			*	6.227 ^a^	1.411 ^c^	2.729 ^b^	1.854 ^c^
4-Ethylphenol	Phenolic compound	107/122/77	1271		Alcohol, medicinal	NS	0.870	1.104	1.546	0.547
Ethyl dihydrocinnamate		104/91	1396	1390		NS	0.469	0.383	nd	nd
β-Bisabolene	Terpene	69/93	1525	1517		NS	0.262	nd	nd	nd
Σ Alcohols						*	1.726 ^b^	2.638 ^a^	1.233 ^b^	2.900 ^a^
Σ Sulfur compounds						NS	0.221	0.552	1.063	0.285
Σ Esters						**	38.48 ^a^	24.44 ^b^	24.64 ^b^	15.78 ^c^
Σ Ketones						**	3.907 ^b,c^	6.412 ^b^	14.47 ^a^	0.974 ^c^
Σ Terpenes						***	27.81 ^c^	29.04 ^b,c^	47.39 ^a^	32.01 ^b^
Σ Acids						*	19.81 ^a^	16.26 ^a^	2.95 ^b^	24.03 ^a^
Σ Phenolic compounds						***	7.000 ^b^	19.14 ^a^	7.488 ^b^	23.50 ^a^
Σ Hydrocarbons						NS	0.580	1.135	0.773	0.523

† NS = not significant at *p* > 0.05. *, ** and *** significant at *p* < 0.05, 0.01, and 0.001, respectively. ‡ Values followed by the same letter within the same row were not significantly different (*p* > 0.05), according to Tukey’s least significant difference test. **^§^** Cano-Lamadrid et al. [2], Angerosa et al. [20], SAFC [22].R.I.: retention index; Exp.: experimental; Lit.: literature; nd: not detected.

**Table 3:** Descriptive sensory attributes of table olives as affected by the irrigation treatment. Scale used ranged from 0 = no intensity to 10 = extremely strong intensity.

	Appearance	Flavor							Texture		
Sample	Color	Green-Olive Flavor	Saltiness	Bitterness	Sourness	Sweetness	Aftertaste	Off-Flavor	Hardness	Crunchiness	Fibrousness
**ANOVA** ^†^											
	**	*	NS	*	***	NS	*	NS	***	***	NS
**Multiple Range Tukey Test** ^‡^											
T0	6.5 ^a,‡^	6.5 ^a,b^	5.9	5.8 ^a^	2.4 ^b^	2.9	5.9 ^a,b^	0.0	7.8 ^a^	7.3 ^a^	0.3
T1	5.4 ^b^	6.9 ^a^	5.0	3.8 ^a,b^	3.0 ^b^	2.1	5.9 ^a,b^	0.0	6.6 ^a^	5.6 ^a^	0.8
T2	5.9 ^a,b^	6.4 ^a,b^	5.9	4.0 ^a,b^	2.6 ^b^	2.2	5.6 ^b^	0.0	7.2 ^a^	6.1 ^a^	0.3
T3	5.7 ^a,b^	6.2 ^b^	4.9	2.8 ^b^	6.9 ^a^	1.7	8.1 ^a^	0.0	3.5 ^b^	1.7 ^b^	0.4

† NS = not significant at *p* > 0.05. *, **, and *** significant at *p* < 0.05, 0.01, and 0.001, respectively. ‡ Values followed by the same letter within the same column were not significantly different (*p* > 0.05), according to Tukey’s least significant difference test.

**Table 4:** Affective sensory analysis (at 3 locations in Spain) of table olives as affected by irrigation treatment.

	Color	Flavor	Bitterness	Saltiness	Sourness	Hardness	Crunchiness	Fibrousness	Aftertaste	Overall Liking
**ANOVA** ^†^										
	NS	NS	NS	NS	NS	NS	NS	NS	NS	NS
**Multiple Range Tukey Test**										
T0	6.2	6.6	6.3	6.2	6.3	7.0	6.7	6.5	6.6	6.5
T1	6.7	6.6	6.3	6.0	6.0	6.7	6.6	6.6	6.2	6.4
T2	6.5	6.3	5.7	6.3	5.8	6.5	6.6	6.5	6.2	6.4
T3	6.5	5.9	5.7	5.9	5.8	6.3	6.5	6.5	5.9	5.7

† NS = not significant at *p* > 0.05.

**Table 5:** Overall liking and satisfaction degree on flavor, saltiness and hardness of Table Olives affected by logo effect and location.

		Green-olive Flavor	Saltiness	Hardness	Overall Liking
**ANOVA Test** ^†^					
Logo effect		***	NS	NS	*
Location		***	NS	NS	*
Logo effect vs Location		***	NS	NS	*
**Multiple Range Tukey Test Logo effect**					
	Conventional	6.7 ^b,‡^	6.4	6.6	6.5 ^b^
	HydroSOStainable logo	8.0 ^a^	7.4	7.0	7.4 ^a^
**Multiple Range Tukey Test Location**					
Location	L1	7.7 ^a^	6.6	6.9	6.9 ^b^
	L2	7.0 ^b^	7.1	7.2	7.3 ^a^
	L3	7.3 ^a,b^	7.0	6.3	6 ^b^
**Multiple Range Tukey Test Logo effect vs. Location**					
Conventional	L1	7.1 ^a,b^	5.9	6.5	6.3 ^a,b^
	L2	7.0 ^a,b^	6.6	7.3	7.6 ^a^
	L3	5.9 ^c^	6.7	5.9	5.6 ^b^
HydroSOStainable logo	L1	8.3 ^a^	7.2	7.3	7.5 ^a^
	L2	6.9 ^b^	7.7	7.0	7.1 ^a,b^
	L3	8.7 ^a^	7.2	6.8	7.7 ^a^

† NS = not significant at *p* > 0.05. *, and ***, significant at *p* < 0.05, and 0.001, respectively. ‡ Values followed by the same letter within the same column and factor (treatment and location) were not significantly different (*p* > 0.05), according to Tukey’s least significant difference test.
